# Supplementary material for: Risk factors for mortality of coronavirus disease-2019 (COVID-19) patients in two centers of Hubei province, China: A retrospective analysis
Source: PLoS One. 2021 Jan 28;16(1):e0246030. doi: 10.1371/journal.pone.0246030 (PMC7842894; doi:10.1371/journal.pone.0246030)
Supplement: S1 Table — (DOCX) [file pone.0246030.s001.docx]

S1 Table. Demography and clinical characteristics of different severity of COVID-19 patients.

| Variable | Total (n=432) | Non-severe (n=307) | Severe (n=125) | *p* value |
| --- | --- | --- | --- | --- |
| Age, years | 54 (39-66) | 50 (36-64) | 62 (48-71) | <0.0001 |
| Gender, n (%) |  |  |  | 0.072 |
| Male | 230 (53.2) | 155 (50.5) | 75 (60.0) |  |
| Female | 202 (46.8) | 152 (49.5) | 50 (40.0) |  |
| Comorbidity, n (%) | 144 (33.3) | 73 (23.8) | 71 (56.8) | <0.0001 |
| Hypertension | 97 (22.5) | 41 (13.4) | 56 (44.8) | <0.0001 |
| ACEI or ARB administration | 20 (20.6) | 10 (24.4) | 10 (17.9) | 0.432 |
| Cardiovascular or cerebrovascular diseases | 25 (5.8) | 9 (2.9) | 16 (12.8) | <0.0001 |
| Diabetes | 56 (13.0) | 25 (8.1) | 31 (24.8) | <0.0001 |
| Chronic obstructive pulmonary disease | 25 (5.8) | 12 (3.9) | 13 (10.4) | <0.0001 |
| Carcinoma | 5 (1.2) | 4 (1.3) | 1 (0.8) | 0.658 |
| Chronic kidney disease | 9 (2.1) | 1 (0.3) | 8 (6.4) | <0.0001 |
| others | 25 (5.8) | 9 (2.9) | 16 (12.8) | <0.0001 |
| Symptom |  |  |  |  |
| Fever | 308 (71.3) | 214 (69.7) | 94 (75.2) | 0.252 |
| Dry cough | 270 (62.5) | 84 (67.2) | 186 (60.6) | 0.198 |
| Fatigue | 128 (29.6) | 46 (36.8) | 82 (26.7) | 0.037 |
| Dyspnea | 35 (8.1) | 15 (4.9) | 20 (16.0) | <0.0001 |
| Sputum production | 130 (30.1) | 80 (26.1) | 50 (40.0) | 0.004 |
| Sore throat | 34 (7.9) | 22 (7.2) | 12 (9.6) | 0.394 |
| Chest tightness | 47 (10.9) | 22 (17.6) | 25 (8.1) | 0.004 |
| Diarrhoea | 20 (4.6) | 14 (4.6) | 6 (4.8) | 0.914 |
| Myalgia | 46 (10.6) | 27 (8.8) | 19 (15.2) | 0.050 |
| Headache | 19 (4.4) | 13 (4.2) | 6 (4.8) | 0.795 |
| Temperature, ℃ | 36.8 (36.5-37.3) | 36.8 (36.5-37.3) | 36.8 (6.6-37.7) | 0.026 |
| Heart rate, beat/min | 88 (80-97) | 88 (80-97) | 88 (80-98) | 0.645 |
| Respiratory rate, breath/min | 21 (20-23) | 20 (20-22) | 21 (20-23) | 0.007 |
| Systolic blood pressure, mmHg | 126 (118-137) | 127 (118-139) | 126 (118-135) | 0.609 |
| Diastolic blood pressure, mmHg | 80 (71-87) | 80 (72-88) | 78 (70-82) | 0.003 |
| Illness onset to hospital admission, days | 5 (2-10) | 4 (2-10) | 5 (3-10) | 0.632 |
| Illness onset to hospital discharge/death, days | 26 (20-36) | 25 （19-34） | 29 （21-39） | 0.014 |
| Number of deaths | 22 (5.1) | 0 (0.0) | 22 (17.6) | <0.0001 |

Data are presented as median (interquartile range) or number (%).

Abbreviations: COVID-19: coronavirus disease-2019; ACEI: angiotensin converting enzyme inhibitors; ARB: angiotensin receptor blocker.
