# Supplementary material for: Risk factors for mortality of coronavirus disease-2019 (COVID-19) patients in two centers of Hubei province, China: A retrospective analysis
Source: PLoS One. 2021 Jan 28;16(1):e0246030. doi: 10.1371/journal.pone.0246030 (PMC7842894; doi:10.1371/journal.pone.0246030)
Supplement: S2 Table — (DOCX) [file pone.0246030.s002.docx]

**S2 Table. Laboratory findings of COVID-19 patients between survivors and non-survivors.**

| **Variable** | **Total (n=432)** | **Non-severe (n=307)** | **Severe (n=125)** | ***p* value** |
| --- | --- | --- | --- | --- |
| White blood cell count, ×10^9^/L | 4.70（3.59-6.10） | 4.60 (3.52-5.91) | 4.80 (3.59-7.25) | 0.146 |
| Lymphocyte count, ×10^9^/L | 1.21 （0.84-1.63） | 1.30 (0.97-1.71) | 0.88 (0.61-1.40) | <0.0001 |
| T cell subsets |  |  |  |  |
| CD4+ T cells, cell/μL | 593 (339-848) | 657 (484-883) | 275 (95-710) | <0.0001 |
| CD8+ T cells, cell/μL | 339 (217-490) | 380 (256-513) | 235 (112-370) | 0.001 |
| Haemoglobin, g/L | 124 (112-136) | 137 (126-147) | 121 (109-134) | 0.010 |
| Platelet count, ×10^9^/L | 158 (119-208) | 162 (123-216) | 148 (106-198) | 0.043 |
| Albumin, g/L | 38.2 (33.9-41.4) | 39.1 (35.8-42.3) | 34.5 (30.7-39.4) | <0.0001 |
| Total bilirubin, μmol/L | 9.30 (6.81-13.15) | 8.81 (6.65-12.00) | 10.40 (7.30-14.96) | 0.002 |
| Direct bilirubin, μmol/L | 2.71 (1.88-3.96) | 2.60 (1.76-3.82) | 3.14 (2.31-4.98) | <0.0001 |
| Alaine aminotransferase, U/L | 22.0 (14.0-35.0) | 21.0 (13.0-31.0) | 25.0 (17.0-42.7) | 0.003 |
| Aspartate aminotransferase, U/L | 22.0 (16.0-30.0) | 20.0 (15.0-28.0) | 25.0 (19.0-37.0) | <0.0001 |
| Creatinine, μmol/L | 68.5 (55.9-82.5) | 67.0 (54.6-79.6) | 73.1 (61.9-90.0) | <0.0001 |
| Cystatin C, mg/L | 1.05 (0.89-1.29) | 1.01 (0.86-1.20) | 1.22 (1.02-1.51) | <0.0001 |
| Creatine kinase-MB, U/L | 8.9 (1.3-12.2) | 8.7 (1.0-11.6) | 9.2 (5.1-13.8) | 0.014 |
| Myoglobin, ng/mL | 36.0 (27.8-79.1) | 33.0 (27.2-55.7) | 59.0 (30.6-95.6) | 0.008 |
| Cardiac troponin I, pg/mL | 4.5 (1.9-11.1) | 2.0 (1.9-8.0) | 10.3 (4.3-21.7) | <0.0001 |
| Brain natriuretic peptide, pg/mL | 104.5 (29.0-311.0) | 54.0 (20.0-191.0) | 270.0 (116.0-672.0) | <0.0001 |
| Creatine kinase, U/L | 62.0 (43.05-98.0) | 59.0 (41.5-89.0) | 75.0 (49.0-131.5) | 0.001 |
| Lactate dehydrogenase, U/L | 206.0 (169.0-265.8) | 193.0 (160.0-238.0) | 260.0 (204.5-358.1) | <0.0001 |
| C reactive protein, mg/L | 12.5 (3.1-38.7) | 9.1 (2.1-26.5) | 28.4 (10.5-72.7) | <0.0001 |
| Erythrocyte sedimentation rate, mm/h | 25.0 (14.5-48.0) | 21.0 (11.0-40.8) | 37.0 (22.0-57.0) | <0.0001 |
| Procalcitonin, ng/mL | 0.07 (0.05-0.11) | 0.06 (0.05-0.09) | 0.09 (0.06-0.18) | <0.0001 |
| Serum lactate, mmol/L | 1.84 (1.49-2.34) | 1.82 (1.29-2.43) | 1.85 (1.55-2.25) | 0.75 |
| Interleukin-2 receptor, U/mL | 413.0 (266.8-697.3) | 368.0 (259.5-603.5) | 725.0 (409.0-922.1) | 0.01 |
| Interleukin-6, pg/mL | 3.5 (1.6-11.9) | 2.7 (1.5-5.4) | 17.1 (4.0-130.3) | <0.0001 |
| Interleukin-8, pg/mL | 9.3 (6.3-12.9) | 7.9 (6.0-11.7) | 11.5 (9.1-21.5) | 0.008 |
| Interleukin-10, pg/mL | 5.0 (5.0-5.0) | 5.0 (5.0-5.0) | 5.0 (5.0-6.6) | 0.031 |
| TNF-α, pg/mL | 7.6 (6.1-9.7) | 7.2 (6.0-9.3) | 8.7 (6.9-13.0) | 0.022 |
| Prothrombin time, s | 11.1 (10.6-12.9) | 11.1 (10.5-13.1) | 11.1 (10.6-12.4) | 0.770 |
| Activated partial thromboplastin time, s | 32.3 (27.7-37.0) | 32.1 (28.2-37.5) | 32.8 (27.5-36.5) | 0.219 |
| D-dimer, μg/ml | 0.54 (0.44-0.80 | 0.52 (0.42-0.63) | 0.62 (0.50-1.42) | <0.0001 |
| PaO_2_, mmHg | 92.0 (76.5-110.0) | 108.0 (95.1-125.0) | 77.8 (66.8-87.0) | <0.0001 |
| PaCO_2_, mmHg | 40.4 (36.9-43.6) | 40.8 (38.0-43.7) | 39.7 (35.0-43.6) | 0.110 |
| PaO_2_: FiO_2_, mmHg | 306.9 (231.8-362.1) | 355.2 (324.5-420.7) | 233.9 (146.7-273.1) | <0.0001 |

^*^: minimum detection value is 5.0.

All data are presented as median interquartile (IQR)

Abbreviations: COVID-19: coronavirus disease-2019; TNF: tumor necrosis factor-α. PaO_2_: arterial partial pressure of oxygen; PaCO_2_: arterial partial pressure of carbon dioxide; FiO_2_: fraction of inspiration O_2_.
