# Supplementary material for: Risk factors for mortality of coronavirus disease-2019 (COVID-19) patients in two centers of Hubei province, China: A retrospective analysis
Source: PLoS One. 2021 Jan 28;16(1):e0246030. doi: 10.1371/journal.pone.0246030 (PMC7842894; doi:10.1371/journal.pone.0246030)
Supplement: S3 Table — (DOCX) [file pone.0246030.s003.docx]

**S3 Table. CT image results of different severity of COVID-19 patients*.**

| **Variable** | **Total (n=416)** | **Non-severe (moderate)** | **Severe** | ***p* value** |
| --- | --- | --- | --- | --- |
| Bilateral lung involved | 343 (82.5) | 223 (76.6) | 120 (96.0) | <0.0001 |
| Ground-glass opacity | 410 (98.6) | 286 (98.3) | 124 (99.2) | 0.471 |
| Consolidation | 97 (23.3) | 56 (19.2) | 41 (32.8) | 0.003 |
| Pleural effusion | 5 (1.2) | 1 (0.3) | 4 (3.2) | 0.014 |
| Pleural thickening | 5 (1.2) | 4 (1.4) | 1 (0.8) | 0.622 |
| CT score | 6 (4-8) | 6 (3-7) | 7 (5-11) | <0.0001 |

*: moderate, severe, and critical cases were included into this analysis.

All data are presented as median interquartile (interquartile range) or number (%).

Abbreviations: CT: computer tomography; COVID-19: coronavirus disease-2019.
