# Supplementary material for: Risk factors for mortality of coronavirus disease-2019 (COVID-19) patients in two centers of Hubei province, China: A retrospective analysis
Source: PLoS One. 2021 Jan 28;16(1):e0246030. doi: 10.1371/journal.pone.0246030 (PMC7842894; doi:10.1371/journal.pone.0246030)
Supplement: S4 Table — (DOCX) [file pone.0246030.s004.docx]

**S4 Table. Treatment and complications between severe and non-severe COVID-19 patients.**

| **Variable** | **Total (n=416)** | **Non-severe** | **Severe*** | ***p* value** |
| --- | --- | --- | --- | --- |
| Antiviral drugs | 417 (96.5) | 297 (96.7) | 120 (96.0) | 0.702 |
| Umifenovir | 237 (54.9) | 166 (54.1) | 71 (56.8) | 0.605 |
| Interferon α nebulization | 265 (61.3) | 180 (58.6) | 85 (68.0) | 0.07 |
| Lopinavir/ritonavir | 257 (59.5) | 177 (57.7) | 80 (64.0) | 0.223 |
| Ribavirin | 29 (6.7) | 18 (5.9) | 11 (8.8) | 0.269 |
| Chloroquine | 25 (5.8) | 22 (7.2) | 3 (2.4) | 0.054 |
| Hydroxychloroquine | 28 (6.5) | 24 (7.8) | 4 (3.2) | 0.077 |
| Oselatmivir | 239 (55.3) | 161 (52.4) | 78 (62.4) | 0.059 |
| Antibiotics | 378 (87.5) | 264 (86.0) | 114 (91.2) | 0.138 |
| Corticosteroids | 106 (24.5) | 41 (13.4) | 65 (52.0) | <0.0001 |
| Intravenous immunoglobulin | 51 (11.8) | 0 (0.0) | 51 (40.8) | <0.0001 |
| Oxygen supply method | 359 (83.1) | 236 (76.9) | 123 (98.4) | <0.0001 |
| Nasal cannula | 299 (69.2) | 234 (76.2) | 65 (52.0) | <0.0001 |
| Nasal and mouth mask | 20 (4.6) | 0 (0.0) | 20 (16.0) | <0.0001 |
| High-flow oxygen therapy | 5 (1.2) | 0 (0.0) | 5 (4.0) | <0.0001 |
| Noninvasive mechanical ventilation | 26 (6.0) | 0 (0.0) | 2 (20.8) | <0.0001 |
| Invasive mechanical ventilation | 6 (1.4) | 0 (0.0) | 6 (4.8) | <0.0001 |
| Continuous renal replacement therapy | 9 (2.1) | 2 (0.7) | 7 (5.6) | 0.001 |
| Complications | 52 (12.0) | 15 (4.9) | 37 (29.6) | <0.0001 |
| ARDS | 24 (5.6) | 0 (0.0) | 24 (19.2) | <0.0001 |
| Acute kidney injury | 16 (3.7) | 7 (2.3) | 9 (7.2) | 0.014 |
| Heart failure | 8 (1.9) | 1 (0.3) | 7 (5.6) | <0.0001 |
| Acute hepatic insufficiency | 30 (6.9) | 14 (4.6) | 16 (12.8) | 0.002 |
| Acute cardiac injury | 3 (0.7) | 0 (0.0) | 3 (2.4) | 0.006 |
| Thrombocytopenia | 9 (2.1) | 2 (0.7) | 7 (5.6) | 0.001 |
| Duration of viral shedding after COVID-19 onset, days | 6 (3-10) | 6 (3-10) | 7 (4-11) | 0.082 |

*: including severe and critical types according to New Coronavirus Pneumonia Prevention and Control Program published by Chinese National Health Committee (version 6).

Abbreviations: COVID-19, coronavirus disease-2019; ARDS, acute respiratory distress syndrome.
