# Supplementary material for: Risk factors for mortality of coronavirus disease-2019 (COVID-19) patients in two centers of Hubei province, China: A retrospective analysis
Source: PLoS One. 2021 Jan 28;16(1):e0246030. doi: 10.1371/journal.pone.0246030 (PMC7842894; doi:10.1371/journal.pone.0246030)
Supplement: S5 Table — (DOCX) [file pone.0246030.s005.docx]

**S5 Table. Normal range of laboratory findings of COVID-19 patients.**

| **Variable** | **Normal range** |
| --- | --- |
| White blood cell count, ×10^9^/L | 4-10 |
| Lymphocyte count, ×10^9^/L | 1.10-3.35 |
| T cell subsets |  |
| CD4+ T cells, cell/μL | 550-1440 |
| CD8+ T cells, cell/μL | 320-1250 |
| Haemoglobin, g/L | 110-150 |
| Platelet count, ×10^9^/L | 100-300 |
| Albumin, g/L | 35-54 |
| Total bilirubin, μmol/L | 2-26 |
| Direct bilirubin, μmol/L | 0-8 |
| Alaine aminotransferase, U/L | 0-40 |
| Aspartate aminotransferase, U/L | 0-40 |
| Creatinine, μmol/L | 35-97 |
| Cystatin C, mg/L | 0.63-1.55 |
| Creatine kinase-MB, U/L | 0-8 |
| Myoglobin, ng/mL | 0-154.9 |
| Cardiac troponin I, pg/mL | 2-35 |
| Brain natriuretic peptide, pg/mL | 0-500 |
| Creatine kinase, U/L | 0-190 |
| Lactate dehydrogenase, U/L | 109-245 |
| C reactive protein, mg/L | 0-10 |
| Erythrocyte sedimentation rate, mm/h | 0-20 |
| Procalcitonin, ng/mL | 0.02-0.5 |
| Serum lactate, mmol/L | 0-2.0 |
| Interleukin-2 receptor, U/mL | 223-710 |
| Interleukin-6, pg/mL | 0-7 |
| Interleukin-8, pg/mL | 0-62 |
| Interleukin-10, pg/mL | 0-9.0 |
| TNF-α, pg/mL | 0-8.1 |
| Prothrombin time, s | 10-13 |
| Activated partial thromboplastin time, s | 23-37 |
| D-dimer, μg/ml | 0-0.5 |
| PaO_2_, mmHg | 80-100 |
| PaCO_2_, mmHg | 35-45 |

Abbreviations: COVID-19: coronavirus disease-2019; TNF: tumor necrosis factor-α. PaO_2_: arterial partial pressure of oxygen; PaCO_2_: arterial partial pressure of carbon dioxide; FiO_2_: fraction of inspired O_2_.
