# Supplementary material for: Risk factors for mortality of coronavirus disease-2019 (COVID-19) patients in two centers of Hubei province, China: A retrospective analysis
Source: PLoS One. 2021 Jan 28;16(1):e0246030. doi: 10.1371/journal.pone.0246030 (PMC7842894; doi:10.1371/journal.pone.0246030)
Supplement: Attachment — Submitted filename: Response to Reviewers.doc [file pone.0246030.s006.doc]

Department of Pulmonary and Critical Care Medicine, Zhongshan Hospital, Xiamen University;

Teaching Hospital of Fujian Medical University;

No.201, Siming District, Xiamen, Fujian Province, 361004, People's Republic of China.

Jan,12^th^, 2021

Re: Manuscript No. PONE-D-20-31483

Title: Risk factors for mortality of coronavirus disease-2019 (COVID-19) patients in two centers of Hubei province, China: a retrospective analysis

Dear Editor Alexandra Lucas,

Thank you for the timely review of our manuscript.

We have attached our point-by-point responses to the reviewers’ and editors’ suggestions and have also responded to the reviewers’ comments in the text by using the ‘track changes’ function in the ‘revised revision’.

This manuscript has been edited and proofread by Medjaden Bioscience Limited.

We hope that the revised manuscript is now acceptable for publication in your journal.

I look forward to hearing from you soon.

Best regards!

Sincerely yours,

Corresponding author: Xiao-Bin Zhang
Email: [zhangxiaobincn@xmu.edu.cn](mailto:zhangxiaobincn@xmu.edu.cn)

**Response to the reviewers and editor's comments**

**Response to the reviewers' comments**

**Reviewer 1 - ACADEMIC EDITOR:**

**Comment 1: Love the paper and like the information but could really benefit from a native English speaker edit**
Response 1: Thank you for your kind comment. Our manuscript has been edited by a native English speaker editor working in a language editing company (Medjaden Incorporate, www.medjaden.com). The language certificate is attached for your reference.

**Comment 2: Line 27--We aim to is awkward-- We analyzed**

Response 2: Thank you for your valuable suggestion. We have re-written this sentence according to your suggestion.

**Comment 3: Line 43- Use standard abbreviation for hemoglobin (Hb) of (Hgb).** 
Response 3: Thank you for your valuable suggestion. The abbreviation for hemoglobin has been changed to Hb in the whole manuscript.

**Comment 4: Line 65- I think you should just change dissimilarity to homology. I thin you misinterpreted the original 'Notably, 2019-nCoV was closely related (with 88% identity) to two bat-derived severe acute respiratory syndrome (SARS)-like coronaviruses, bat-SL-CoVZC45 and bat-SL-CoVZXC21, collected in 2018 in Zhoushan, eastern China, but were more distant from SARS-CoV (about 79%) and MERS-CoV (about 50%).'**

Response 4: Thank you for your valuable comment. We apologized for our mistake. The relevant context has been corrected according to your comment as follows: “Being a beta coronavirus, the SARS-CoV-2 virus was closely related (with 88% identity) to two bat-derived severe acute respiratory syndrome (SARS)-like coronaviruses, bat-SL-CoVZC45 and bat-SL-CoVZXC21, collected in 2018 in Zhoushan, eastern China, but were more distant from SARS-CoV (about 79%) and MERS-CoV (about 50%)”.

**Comment 5: Line 80- the tense is odd..Instead of We tried. ...We provide these things continue but as above-just a good edit**

Response 5: Thank you for your valuable suggestion. This manuscript has been edited and proofread by Medjaden Bioscience Limited to ensure that it is readable.

**Comment 6: Line 118. is it CK or CK-MB?  Would also include normal ranges for all measurements**

Response 6: Thank you for your thoughtful question and valuable suggestion. Both CK and CK-MB were analyzed in our manuscript. The normal ranges for all measurements are outlined in eTable 5.

**Reviewer 2 Comments**

Comment 1: Zhang et al. report clinical characteristics of 432 COVID-19 patients from two hospitals in Wuhan, China. The authors stratify their analyses by survivors (n=410) and non-survivors(n=22). Using multivariate Cox regression analyses, the authors determined that hemoglobin, creatine kinase-MB, lactate dehydrogenase and procalcitonin levels are predictors of COVID-19 mortality. This is consistent with similar published reports. **Small number of non-survivors group is a limitation of the study, not acknowledged in discussion**. Nonetheless, the data on the survivors group is informative and worth publishing. Typically, **predictive models using the identified risk factors are presented in these studies (reviewer does not think this is needed for acceptance, but that would be helpful).**

Response 1: Thank you for your positive feedback. We have acknowledged that our manuscript has several limitations, such as retrospective design, lack of several biochemical markers, and small number of patients in the non-survivor group. The small number of patients in the non-survivor group of our study has been included as a limitation in the Discussion section of the revised manuscript. Since we analyzed the predictive factors of survival in COVID-19 patients, Cox regression was considered the first choice (J Allergy Clin Immunol. 2020 Jul;146(1):110-118).

**Authors should address the following:
Comment 2. Please provide details on laboratory testing methodologies (lines 115-122).**

Response 2: Thank you for your valuable suggestion. The methodologies of all laboratory tests have been presented in the “Methods” section of the revised manuscript.

**Comment 3. Please clarify “ref” values in Table 5.**
Response 3: Thank you for your thoughtful comment. The “ref” value in Table 5 is 1.0 (*Lancet 2020; 395:1054-1062*). This issue has been corrected in the revised manuscript.

**Response to the editor's comments**

**Comment 1. Please ensure that your manuscript meets PLOS ONE's style requirements, including those for file naming. The PLOS ONE style templates can be found at**

**https://journals.plos.org/plosone/s/file?id=wjVg/PLOSOne_formatting_sample_main_body.pdf and**

**https://journals.plos.org/plosone/s/file?id=ba62/PLOSOne_formatting_sample_title_authors_affiliations.pdf**

 Response 1: Thank you for your valuable comment. The manuscript has been modified according to the journal style.

**Comment 2.  Thank you for stating the following in the Acknowledgments Section of your manuscript:**

**" The funders had no role in study design, data collection and analysis, decision to publish, or preparation of the manuscript."**

**We note that you have provided funding information that is not currently declared in your Funding Statement. However, funding information should not appear in the Acknowledgments section or other areas of your manuscript. We will only publish funding information present in the Funding Statement section of the online submission form.**

**Please remove any funding-related text from the manuscript and let us know how you would like to update your Funding Statement. Currently, your Funding Statement reads as follows:**

**"This work was supported by Grant 2018-2-65 for Youth Research Fund
 from Fujian Provincial Health Bureau, and Grant 2018J01393 for Fund from Natural
Science Foundation of Fujian Province, China."**

**Please include your amended statements within your cover letter; we will change the online submission form on your behalf.**

 Response 2: Thank you for your valuable suggestion. The Funding information has been deleted from the Acknowledgements section and it has not been presented in any other sections of our manuscript. Meanwhile, we have included the Funding information in the cover letter as follows: “This work was supported by Grant 2018-2-65 for Youth Research Fund from Fujian Provincial Health Bureau, Grant 2020GGB057 for Young people training project from Fujian Province Health Bureau, and Grant 2018J01393 for Fund from Natural Science Foundation of Fujian Province, China.”

**Comment 3. Your ethics statement should only appear in the Methods section of your manuscript. If your ethics statement is written in any section besides the Methods, please move it to the Methods section and delete it from any other section. Please ensure that your ethics statement is included in your manuscript, as the ethics statement entered into the online submission form will not be published alongside your manuscript.**

Response 3: Thank you for your valuable suggestion. Our ethics statement only appears in the “Methods” section of the revised manuscript.

***We would like to take this opportunity to express our gratitude and appreciation for the meticulous and professional comments provided by the reviewers and editors that have contributed greatly to improving our work.***
